# Editorial: Physical exercise and diabetes: exploring the relationship and impact on health outcome

**DOI:** 10.3389/fendo.2024.1505709

**Published:** 2024-10-17

**Authors:** Roberto Codella, Iván Chulvi-Medrano, Fernando Martin-Rivera

**Affiliations:** ^1^ Department of Biomedical Sciences for Health, Università degli Studi di Milano, Milan, Italy; ^2^ Department of Endocrinology, Nutrition and Metabolic Diseases, IRCCS MultiMedica, Milan, Italy; ^3^ Research Group on Prevention and Health in Exercise and Sport (PHES), Department of Physical and Sports Education, University of Valencia, Valencia, Spain

**Keywords:** type 2 diabetes, exercise-as-a-medicine, training, metabolism, health

Type 2 diabetes mellitus (T2DM) has reached pandemic proportions globally, affecting over 400 million people. Beyond the well-known metabolic dysfunctions, such as impaired glucose regulation and insulin resistance, T2DM is closely linked to a plethora of comorbidities (1), result in severe long-term complications including cardiovascular disease, nonalcoholic fatty liver disease (NAFLD), stroke, neuropathy and nephropathy. Research has increasingly underscored the critical role of physical activity in managing T2DM, improving metabolic control, and enhancing overall quality of life. Recent studies have further illuminated the potential of varied exercise modalities to offer targeted benefits across different metabolic, cardiovascular, and muscular systems (2).

This Research Topic presents a collection of 10 insightful articles exploring how various forms of exercise influence the management of T2DM and its related complications, offering new avenues for treatment and rehabilitation.

A key study by Chen et al. explored the efficacy of different exercise interventions in individuals with both T2DM and a history of stroke, focusing on glycemic regulation, lipid profiles, and functional recovery. The study compared low-to-moderate intensity continuous training (LMICT), moderate-intensity interval training (MIIT), and reduced-exertion high-intensity training (REHIT) over a month-long intervention. MIIT and REHIT yielded significant improvements in glycemic control, as measured by continuous glucose monitoring (CGM) metrics, with notable reductions in glucose variability and time above target ranges. These findings align with the growing consensus that interval-based exercise protocols, particularly those involving moderate to high intensity, can be especially beneficial in reducing blood glucose fluctuations in diabetic patients. Furthermore, the REHIT group demonstrated improved functional recovery, which may offer a dual benefit in patients recovering from stroke.

The modest effects on lipid profiles seen across all exercise groups suggest that while glycemic control may be responsive to short-term interventions, longer or more targeted strategies may be needed to influence lipid metabolism. Nonetheless, the potential of MIIT and REHIT to simultaneously manage glucose variability and enhance functional outcomes is promising, particularly for complex cases involving comorbidities like stroke.

In the context of NAFLD, a common comorbidity in T2DM, Haxhi et al. investigated the effects of a counseling intervention aimed at reducing sedentary time and increasing physical activity (PA) in diabetic patients. Over a three-year period, even modest increases in moderate-to-vigorous physical activity (MVPA) were associated with significant reductions in NAFLD markers, including liver enzymes and indices of hepatic steatosis. Importantly, the intervention’s success was driven not solely by achieving high levels of MVPA but by reducing sedentary time and increasing light-intensity activity.

These findings challenge the traditional emphasis on high volumes of vigorous exercise in NAFLD management and suggest that comprehensive lifestyle changes, even if modest, can positively impact liver health. For individuals with T2DM, particularly those with limited mobility or exercise capacity, this approach offers a feasible alternative to structured, high-intensity exercise regimens.

Ma et al. examined the efficacy of blood flow-restricted resistance (BFR) training compared to moderate-intensity resistance training (RT) in improving metabolic health and body composition in older adults with T2DM. Over six months, both exercise groups saw significant improvements in fasting plasma glucose (FPG), HbA1c, and blood lipid levels. Interestingly, BFR was found to be equally effective as moderate-intensity RT in enhancing these metabolic markers, despite involving lower exertion levels.

For older adults, particularly those new to exercise, BFR offers a promising alternative that minimizes strain and mechanical load while delivering similar metabolic benefits. The potential to improve muscle performance and body composition without necessitating high exertion makes BFR an attractive option for individuals with mobility issues or those recovering from injury.

The study by Yang et al. provided a comprehensive analysis of the global burden of T2DM attributable to physical inactivity. Between 1990 and 2019, mortality and disability-adjusted life years (DALYs) due to physical inactivity more than doubled. This alarming trend underscores the need for public health interventions aimed at increasing PA levels worldwide. Notably, countries with lower socioeconomic indices experienced a disproportionately higher burden of inactivity-related T2DM, highlighting the need for equitable access to exercise facilities and public health programs.

This global perspective reinforces the role of physical inactivity as a key modifiable risk factor in the prevention and management of T2DM. Addressing the disparities in PA levels across different regions and socioeconomic groups will be crucial in curbing the global diabetes epidemic.

A fascinating dimension of exercise’s role in T2DM management involves its effects on inflammation. Kim et al. explored the relationship between exercise and serum polyclonal free light chains (FLCs), a biomarker of immune activation and inflammation. After nine months of aerobic, resistance, or combined exercise, T2DM patients exhibited reduced levels of FLCs, suggesting a protective effect against chronic low-grade inflammation.

Notably, this reduction in FLCs occurred regardless of exercise modality, indicating that the anti-inflammatory benefits of exercise are not modality-specific but rather a general consequence of increased PA. Given the role of chronic inflammation in the pathogenesis of T2DM and its complications, FLCs may emerge as a valuable biomarker for monitoring the effectiveness of exercise interventions in mitigating inflammatory states.

Yang et al. investigated the potential mediating role of remnant cholesterol (RC) in the relationship between PA and diabetes risk. Their findings revealed that higher levels of PA were associated with a lower risk of T2DM, with RC acting as a partial mediator. This study adds a novel layer to our understanding of how exercise impacts lipid metabolism and diabetes risk.

The identification of RC as a mediator suggests that PA may reduce T2DM risk not only through traditional mechanisms like improved insulin sensitivity but also by favorably altering lipid metabolism. This highlights the multifaceted benefits of PA in metabolic health.

Lastly, Qiu et al.‘s meta-analysis examined the impact of exercise on flow-mediated dilation (FMD) in T2DM patients. High-intensity interval training (HIIT) emerged as the most effective exercise modality for improving FMD, a marker of vascular health. Frequent, shorter exercise sessions (<60 minutes) were associated with the most significant improvements in FMD.

These findings provide strong evidence for recommending HIIT as a superior strategy for enhancing vascular function in T2DM patients, potentially offering protection against cardiovascular complications.

The emerging research highlights the diverse and far-reaching benefits of exercise in managing T2DM and its associated complications. From glycemic control and lipid metabolism to inflammation and vascular health, the evidence is clear: regular physical activity, regardless of intensity or modality, plays a central role in improving outcomes for individuals with T2DM. As research continues to evolve, a personalized, multidimensional approach to exercise may emerge as the gold standard for diabetes care.
